# Visual representation bias in artificial intelligence–generated depictions of anesthesia, pain, and intensive care professionals

**DOI:** 10.1177/03000605261470625

**Published:** 2026-08-31

**Authors:** Muzeyyen Beldagli, Ahmet Ozan Aydin, Mehmet Alperen Avci, Selma Kahyaoglu, Taner Tunc, Yavuz Yigit, Serkan Tulgar

**Affiliations:** 1 Department of Anesthesiology and Reanimation, Samsun City Hospital, Turkiye; 2 Department of General Surgery, Faculty of Medicine, 653321 Samsun University, Turkiye; 3 Department of Anesthesiology and Reanimation, Faculty of Medicine, Bahçeşehir University, Turkiye; 4 Department of Anesthesiology and Reanimation, Göztepe Medical Park Hospital, Turkiye; 5 Department of Statistics, Faculty of Science, 37139 Ondokuz Mayıs University, Turkiye; 6 Department of Emergency Medicine, 36977 Hamad Medical Corporation, Qatar; 7 Blizard Institute, Queen Mary University of London, United Kingdom

**Keywords:** Artificial intelligence, text-to-image models, anesthesiology, pain medicine, sex representation, ethnic diversity, medical illustration

## Abstract

**Objective:**

To evaluate the demographic and hierarchical representation biases in artificial intelligence–generated depictions of healthcare professionals in anesthesiology, pain medicine, and intensive care, with a focus on sex, age group, skin tone, ethnicity, and professional role hierarchy.

**Methods:**

This cross-sectional comparative study analyzed 4400 artificial intelligence–generated images produced by 4 text-to-image models (DALL·E 3, Midjourney, Leonardo AI, and Gemini). Ten professional roles in anesthesiology, pain medicine, and intensive care, ranging from trainees to department heads, were evaluated. Two independent anesthesiologists with clinical experience in anesthesiology and intensive care assessed each image using a structured digital evaluation form, recording sex, age group, skin tone, ethnicity, and professional role hierarchy. Statistical comparisons were performed using chi-square test with Bonferroni-adjusted post hoc proportion analyses.

**Results:**

Male representation predominated across roles and models (mean: 68.5%), with leadership positions showing the highest male proportion (up to 90.0%). Light skin tones (mean: 70.6%) and Caucasian ethnicity (mean: 68.0%) were the most commonly depicted categories. Younger individuals (age: <40 years) were overrepresented (mean: 53.1%), whereas individuals aged >60 years were rarely depicted (<3.0%) and appeared mainly in leadership roles. Significant differences in sex representation were observed in 9 out of 10 professional roles across artificial intelligence models, with the exception of the anesthesia specialist role (*p* = 0.051).

**Conclusions:**

Text-to-image artificial intelligence systems consistently reproduced results with demographic and hierarchical biases in depictions of healthcare professionals in anesthesiology, pain medicine, and intensive care. The predominance of male, light skin tone, and Caucasian ethnicity, particularly in senior roles, highlights the need for more diverse training datasets and greater transparency in the development of artificial intelligence systems.

## Introduction

Text-to-image artificial intelligence (AI) tools have quickly integrated into many aspects of healthcare practice.^1^ They are now used to produce educational resources, design patient information material, create visual content for scientific presentations, and support broader health communication.^1,2^ By interpreting written prompts, these systems can generate images that are often highly realistic and contextually tailored. Despite these advantages in efficiency and accessibility, concerns have been raised about systematic biases in their output, particularly in depictions of sex, ethnicity, and age. These biases may influence public perception and reinforce existing stereotypes.^3^

Text-to-image AI systems are increasingly used to create medical illustrations and educational visuals.^2,4^ Previous studies across several medical specialties, including anesthesiology, cardiology, and emergency medicine, have reported demographic imbalances in AI-generated depictions of healthcare professionals, particularly with respect to sex and ethnicity.^5–7^ However, most studies have focused on single specialties or a limited range of demographic characteristics. Limited information is available regarding whether these biases also extend to professional hierarchy and occupational roles, such as residents, specialists, technicians, and department heads. The fields of anesthesiology, pain medicine, and intensive care were selected for the present study because they represent closely related yet distinct clinical domains characterized by hierarchical professional structures and multidisciplinary collaboration. A comprehensive evaluation of these interconnected specialties may provide a broader understanding of demographic and hierarchical representation patterns in clinically relevant healthcare environments.

In this context, the aim of the present study was to evaluate demographic and hierarchical representation patterns in AI-generated depictions of healthcare professionals across anesthesiology, pain medicine, and intensive care.

## Materials and methods

### Study design and ethical considerations

A cross-sectional comparative study was conducted to evaluate the demographic and hierarchical representation in AI-generated images of healthcare professionals. Four widely used text-to-image AI models were assessed: (a) DALL·E 3 (OpenAI); (b) Midjourney (Midjourney Inc.); (c) Leonardo AI (Leonardo Interactive Pty Ltd); and (d) Gemini (Google DeepMind). All image generation and analysis were performed digitally between July and August 2025. All images were generated by AI systems in response to standardized textual prompts. We used the Strengthening the Reporting of Observational Studies in Epidemiology (STROBE) guideline to prepare this manuscript, and the completed STROBE checklist is provided as Supplementary Material S1.^8^ Ethical approval was obtained from the Samsun University Clinical Research Ethics Committee (Approval Number: GOKAEK 2025/14/4).

### Image generation

The study included the three closely related medical domains of anesthesiology, pain medicine, and intensive care and 10 predefined professional roles representing different levels of professional hierarchy within each domain, ranging from trainees to senior leadership positions. The predefined roles included anesthesiologist, anesthesiology resident, anesthesia technician, pain specialist, pain fellow, intensive care specialist, intensive care fellow, head of the anesthesiology department, head of the pain department, and head of the intensive care department. For each role, both individual and group-image prompts were generated. Each role was represented by 100 single and 10 group images, with each group depicting 10 individuals, resulting in a total of 4000 individual and 400 group images.

All prompts were standardized in English and specified only the professional role and number of individuals without reference to sex, ethnicity, age, or other demographic characteristics to minimize prompt-induced bias. Representative examples of individual and group prompts (e.g. “A photo of an anesthesiologist” and “A photo of ten anesthesiologists”), the corresponding generated outputs, and the complete prompt list are provided in Supplementary Material S2.

No preparatory prompts were used prior to image generation, and all models were prompted directly using the predefined standardized prompts. Images were generated using standardized prompts applied in a single-shot manner without iterative refinement or feedback-based modification. The same user account was used throughout the study; however, the browser page was refreshed before each generation to ensure that previous outputs did not influence subsequent results.

All generated images were included in the analyses without subjective selection or exclusion. Image generation was performed using DALL·E 3, Midjourney v6, Leonardo AI, and Gemini (all July 2025 versions). All generated images were archived for subsequent evaluation. The AI platforms were used solely for image generation, and no AI tools were used for data analyses or interpretation.

### Image assessment

All images were independently reviewed by two anesthesiologists using a structured digital evaluation form developed specifically for this study. Reviewers recorded the apparent sex (female, male, or unclear); age group (<40, 40–60, or >60 years); skin tone (scored as numeric values obtained using the 10-point Massey–Martin Skin Color Scale and subsequently categorized as light (1–3), medium (4–7), or dark (8–10)) and ethnicity (Caucasian or non-Caucasian).^9^ Both reviewers had clinical and academic experience in anesthesiology, pain medicine, and intensive care as well as prior experience in visual content analysis. In group images, each depicted individual was evaluated separately using the same criteria applied to individual images. Any disagreements between reviewers were resolved through discussion with a third researcher.

Sex was determined based on visual presentation and clothing style, and the category “unclear sex” was assigned when visual cues were insufficient for reliable classification. Age group was estimated based on facial and physical features. Ethnicity was classified based on observable phenotypic traits and cultural indicators and categorized as Caucasian or non-Caucasian. In this study, the term “Caucasian” was used as a descriptive category based on visible phenotypic characteristics rather than as a biological or genetic classification. Classification decisions were based on predefined visual criteria to improve classification consistency across images and reduce subjective variability.

### Statistical analyses

All data recorded in the structured digital evaluation form were transferred to a predesigned spreadsheet and analyzed using the Statistical Package for the Social Sciences (SPSS) software (version 21, IBM Corp.; Armonk, NY, USA). Descriptive statistics were calculated as counts and percentages for categorical variables. Comparisons between AI models and professional roles were performed using chi-square tests. When appropriate, differences in proportions between AI models were assessed using post hoc proportion tests with Bonferroni correction. Statistical significance was defined as a *p*-value <0.05.

## Results

**Figure 1.**
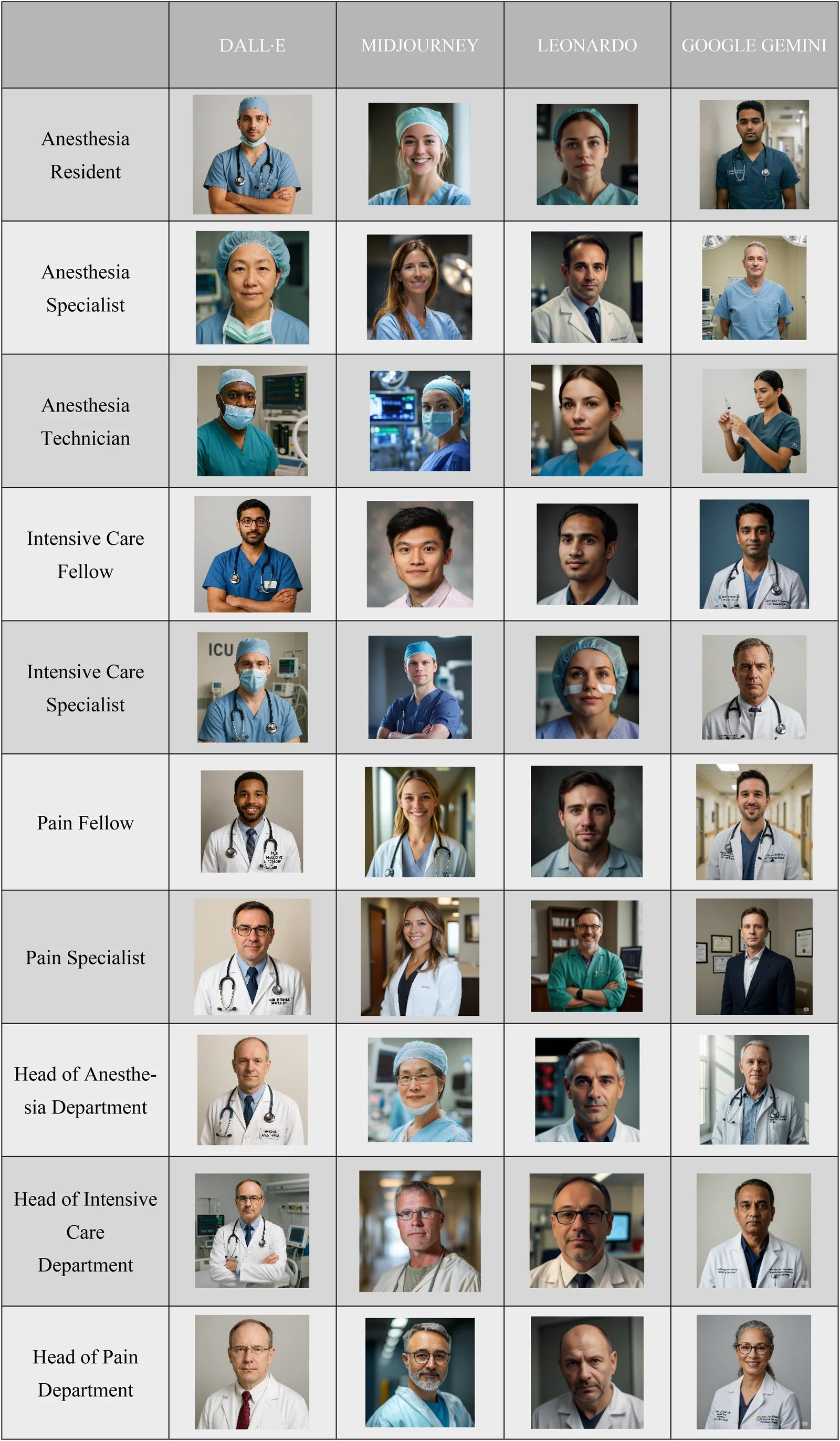
Representative AI-generated depictions of healthcare professionals across 10 predefined roles and four text-to-image models (DALL·E, Midjourney, Leonardo, and Gemini). Images illustrate commonly observed demographic patterns across roles and models.

In total, 4400 AI-generated images were included in the final analysis. Overall inter-rater agreement between reviewers was >95%. Representative examples reflecting the predominant demographic patterns observed in the dataset were selected by the authors (Figure 1).

AI: artificial intelligence.

### Sex distribution

**Figure 2.**
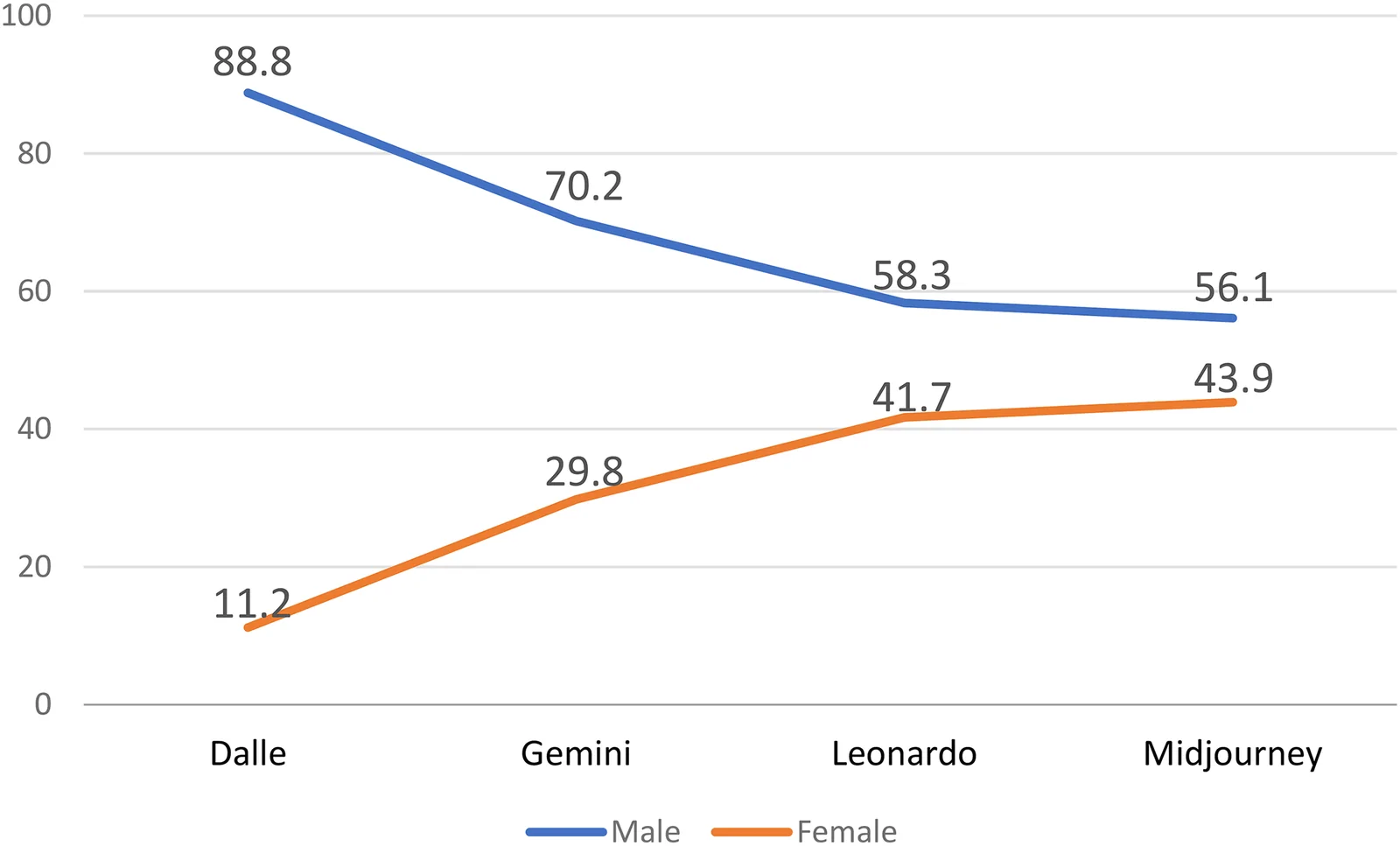
Sex distribution across professional roles generated by four text-to-image models (DALL·E, Gemini, Leonardo, and Midjourney). Values are presented as percentages.

**Figure 3.**
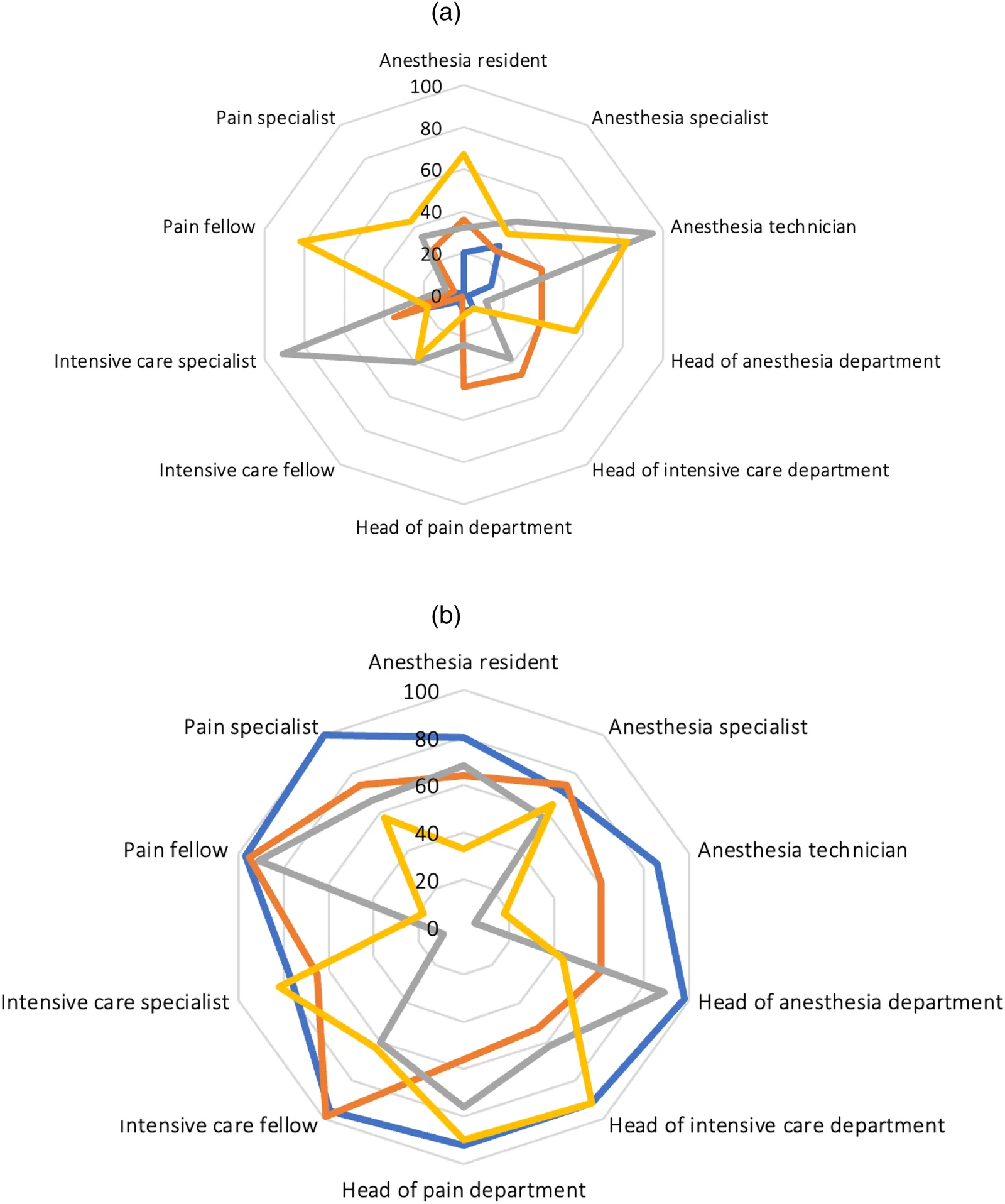
Distribution of male and female representations across 10 professional roles by AI model. (a) Female depictions and (b) male depictions. The radar plots illustrate sex representation across anesthesiology, pain medicine, and intensive care roles. Each colored line represents one AI model (blue, DALL·E 3; orange, Gemini; gray, Leonardo AI; and yellow, Midjourney). Values represent the percentage of depictions classified as (a) female or (b) male.

Across all roles and models, male representation predominated (mean: 68.5%, range 56.1%–88.8%) compared with female representation (mean: 31.8%, range 11.2%–43.9%) (Table 1, Figure 2). Leadership positions, such as heads of departments, consistently displayed the highest male proportions (up to 90%). With respect to the anesthesia technician role, sex distribution differed significantly across AI models (*p* < 0.001), with Leonardo generating mostly female individuals (95%) and DALL·E mostly male individuals (86%) (Figure 3(a) and (b), Table 1). In the individual (single-image) analysis, statistically significant sex differences were observed in almost all roles (*p* < 0.001 for 9 out of 10 roles), except for the anesthesia specialist role (*p* = 0.051). The models displayed notable differences in female-to-male ratios, with Leonardo frequently generating a higher proportion of female characters, particularly for the roles of anesthesia technician, head of the intensive care department, and intensive care fellow.

AI: artificial intelligence.

**Table 1.** Values are expressed as percentages of the total representations for each role.

Staff	Sex (%)								χ^2^	*p*-value
	Female				Male					
	DALL·E	Gemini	Leonardo	Midjourney	DALL·E	Gemini	Leonardo	Midjourney		
Anesthesia resident	20^a^	36^a^	32^a^	67^b^	80^a^	64^a^	68^a^	33^b^	50.575	<0.001
Anesthesia specialist	29^a^	26^a^	43^a^	36^a^	71^a^	74^a^	57^a^	64^a^	7.766	0.051
Anesthesia technician	14^a^	39^b^	95^c^	82^d^	86^a^	61^b^	5^c^	18^d^	173.545	<0.001
Head of anesthesia department	2^a^	39^b^	11^a^	56^b^	98^a^	61^b^	89^a^	44^b^	94.673	<0.001
Head of intensive care department	8^a^	47^b^	38^b^	8^a^	92^a^	53^b^	62^b^	92^a^	65.207	<0.001
Head of pain department	8^a^	44^b^	24^c^	10^a,c^	92^a^	56^b^	76^c^	90^a,c^	49.000	<0.001
Intensive care fellow	4^a^	1^a^	40^b^	37^b^	96^a^	99^a^	60^b^	63^b^	80.074	<0.001
Intensive care specialist	24^a,b^	35^b^	91^c^	18^a^	76^a,b^	65^b^	9^c^	82^a^	137.521	<0.001
Pain fellow	3^a^	5^a^	9^a^	82^b^	97^a^	95^a^	91^a^	18^b^	235.645	<0.001
Pain specialist	0^a^	26^b^	34^b^	43^b^	100^a^	74^b^	66^b^	57^b^	53.807	<0.001

Different superscript letters (a, b, c, d) denote statistically significant differences between AI models in the same row (*p* < 0.05, Bonferroni-adjusted χ^2^ test). Identical letters indicate no significant difference.

### Age distribution

**Figure 4.**
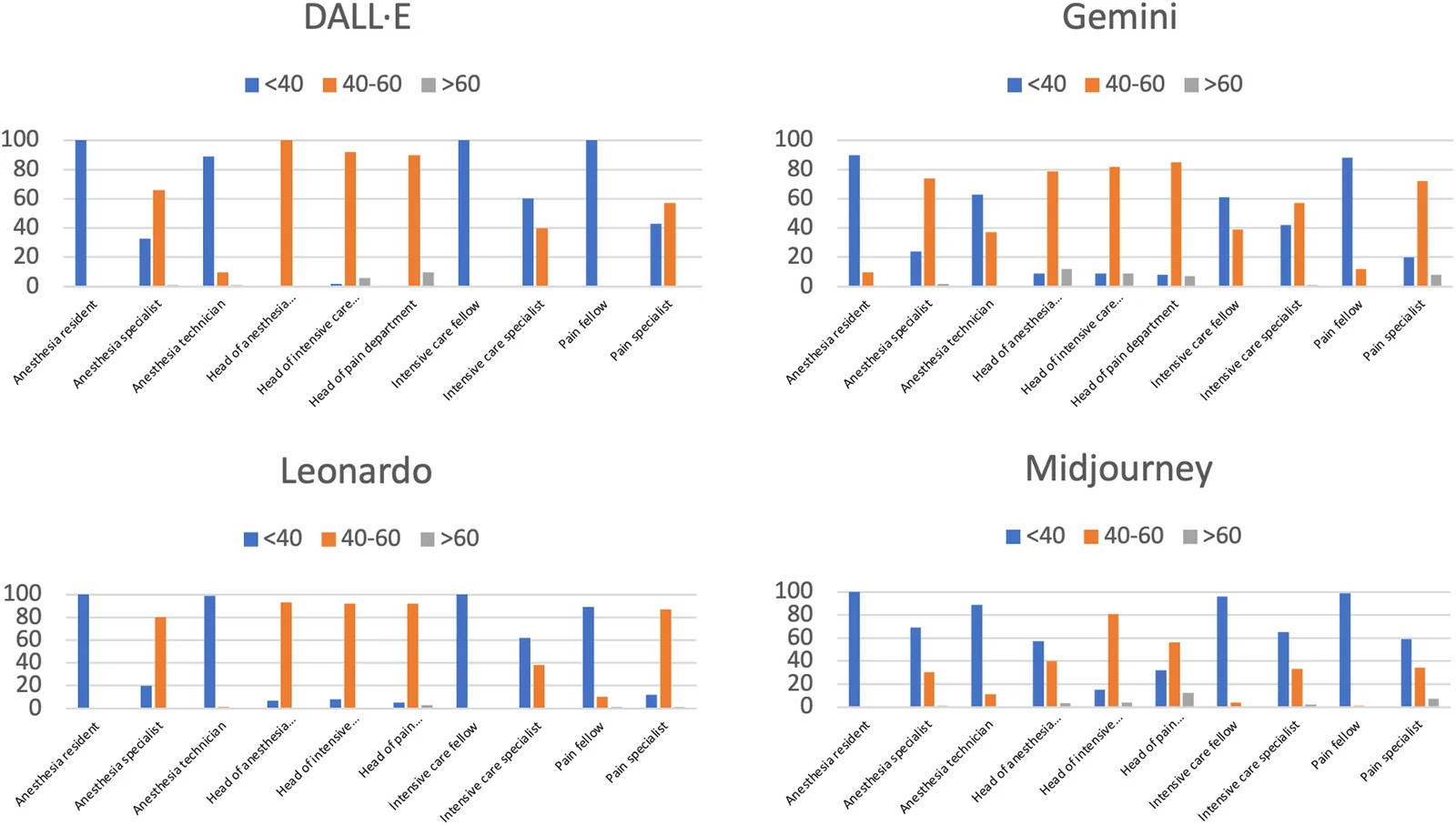
Distribution of age categories across professional roles and AI models generated by four text-to-image systems (DALL·E 3, Gemini, Leonardo AI, and Midjourney). Age groups were defined as <40, 40–60, and >60 years. Values represent the percentages of depictions within each role.

Most individuals were depicted as <40 years of age (mean: 53.1%, range: 41.4%–68.1%), 29%–54.4% as 40–60 years old, and <3% as >60 years old (Figure 4). The youngest individuals were typically depicted in trainee or junior roles, whereas older individuals were more commonly depicted in leadership roles.

AI: artificial intelligence.

### Skin tone distribution

**Figure 5.**
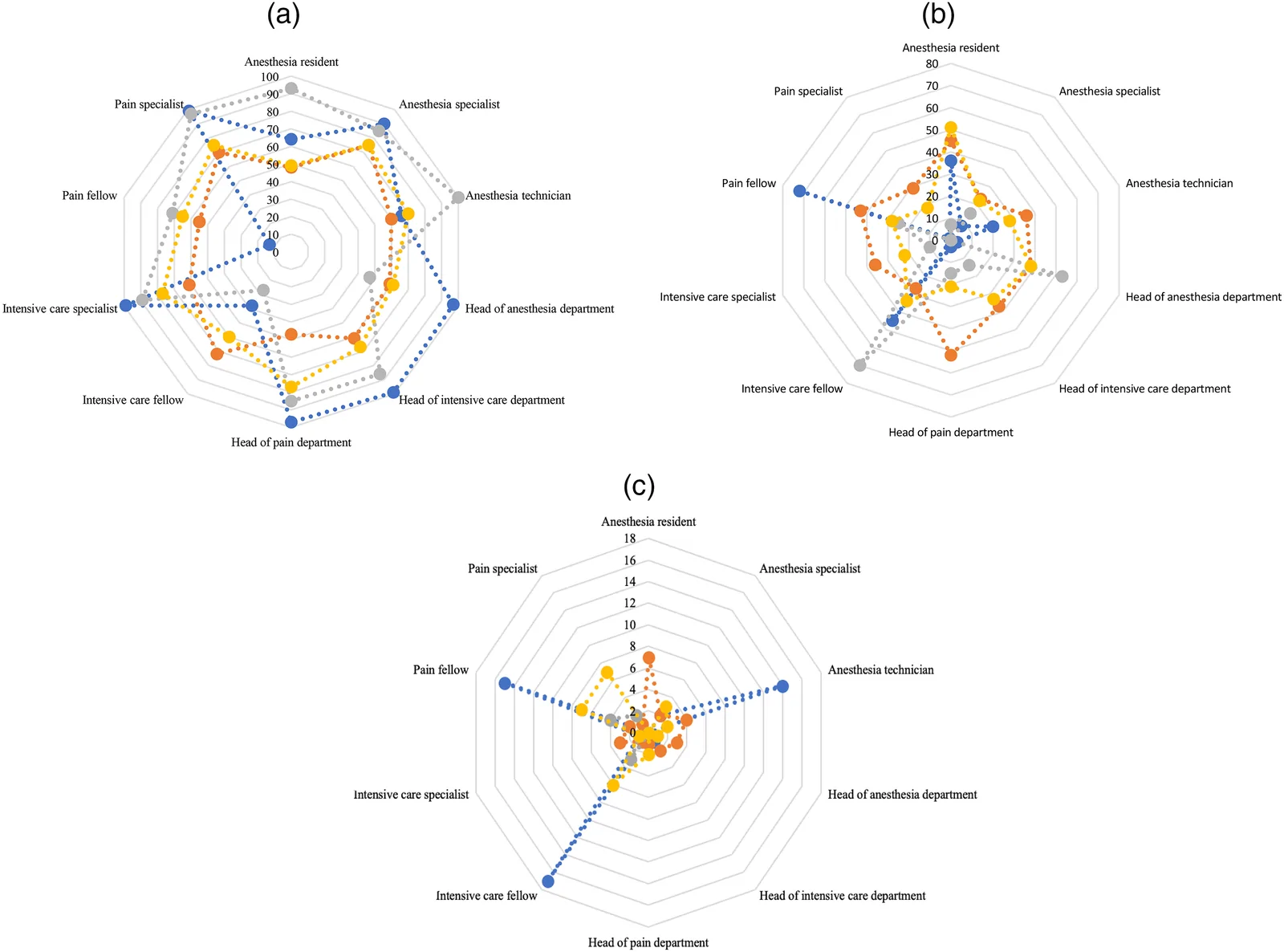
Distribution of skin tone categories across 10 professional roles as generated by AI models. (a) Light skin tones; (b) medium skin tones; and (c) dark skin tones. Radar plots show the proportional distribution of skin tone categories across anesthesiology, pain medicine, and intensive care roles. Each colored line represents one AI model (blue, DALL·E 3; orange, Gemini; gray, Leonardo AI; and yellow, Midjourney). Values represent percentages of depictions within each role.

Light skin tones were the dominant category across all professional roles and AI models (mean: 70.7%, range: 60.8%–78%), followed by medium tones (mean: 26.5%) and dark tones (mean: 2.9%) (Figure 5(a) to (c)). Light skin predominance was most evident in senior and leadership positions, which were almost exclusively depicted as having lighter complexions. DALL·E and Midjourney produced the highest proportion of images depicting individuals with light skin tones, whereas Leonardo showed slightly greater variability across roles. Dark skin tones were rarely represented across all models, indicating overall underrepresentation of darker skin tones.

AI: artificial intelligence.

### Ethnicity distribution

**Figure 6.**
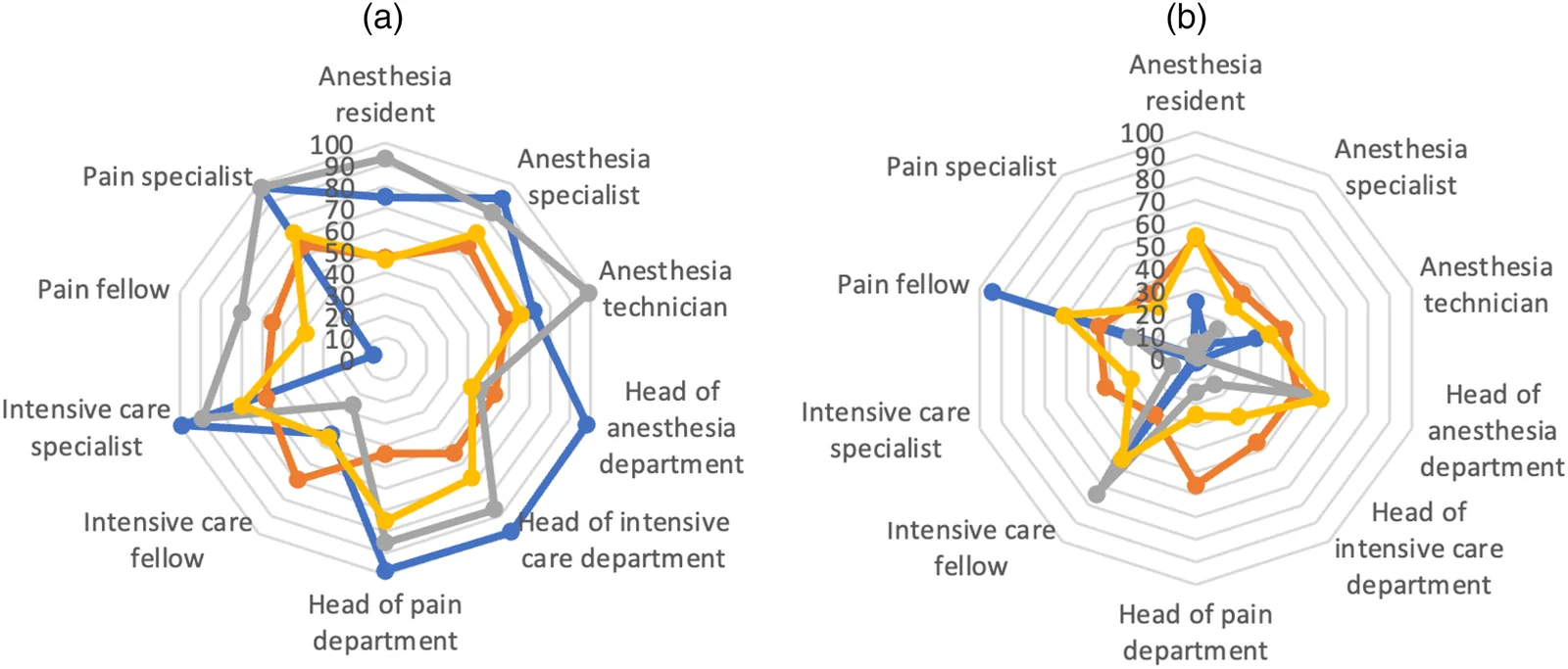
Distribution of ethnicity categories across 10 professional roles as generated by AI models. (a) Caucasian representations and (b) non-Caucasian representations. Radar plots show the proportional distribution of ethnicity groups across anesthesiology, pain medicine, and intensive care roles. Each colored line represents one AI model (blue, DALL·E 3; orange, Gemini; gray, Leonardo AI; and yellow, Midjourney). Values represent percentages of depictions within each role.

Caucasian ethnicity was most frequently represented (mean: 68%, range: 56.9%–78%), with non-Caucasian ethnicities comprising the remaining proportion (Figure 6(a) and (b)). Similar to the observation for skin tone representation patterns, leadership roles showed consistently greater Caucasian representation, which exceeded 80% in several models. As shown in Figure 5, DALL·E and Midjourney produced the most homogeneous outputs dominated by Caucasian representation, whereas Leonardo and Gemini showed slightly greater variability but still limited diversity overall.

AI: artificial intelligence.

### Group images

A supplementary analysis using images generated in groups of 10 was conducted, and the detailed statistical outcomes are presented in the Supplementary Material S3. Compared with individual-image results, grouped generations showed reduced variability among models. For example, sex distributions in grouped images approached balance in some models, such as Midjourney (female: 50.1% and male: 49.5%), whereas others, including Gemini (male: 64.3% and female: 35.6%), remained more male-dominant.

Several differences previously observed in the single-image analysis decreased in the grouped (10-image) analysis, although statistically significant inter-model differences persisted in depictions of anesthesia technician (*p* < 0.001), head of the anesthesia department (*p* = 0.042), head of the intensive care department (*p* < 0.001), head of the pain department (*p* = 0.001), intensive care fellow (*p* < 0.001), and intensive care specialist (*p* < 0.001).

In the grouped analysis, sex imbalance decreased in several roles, including anesthesia resident and head of the anesthesia department, although balanced proportions were not fully achieved. In the anesthesia technician and intensive care fellow roles, female predominance remained noticeable in Leonardo-generated images. By contrast, Midjourney-generated group images showed nearly balanced sex distributions, whereas DALL·E and Gemini continued to exhibit higher male representation. In pain specialist and pain fellow roles, categories that were female-dominant in single-image analyses appeared more balanced or slightly male-predominant in group-image analysis, suggesting a change in representation patterns. However, male predominance remained evident in managerial and technically demanding roles, particularly head and technician positions.

Light skin tones remained the predominant category across all professional roles and AI models, although the inter-model variability decreased compared with single-image generations. Medium tones appeared slightly more common in images generated by Gemini and Leonardo, whereas dark tones remained consistently underrepresented in all models. None of the professional roles demonstrated a predominance of darker skin tones. Detailed numerical results and chi-square statistics for the grouped analysis are provided in the Supplementary material S3.

In the group-image (10-image) analysis, the age distribution pattern was generally similar to that observed in the single-image results, although some differences were noted. Most individuals were still depicted as <40 years old, whereas representations of those aged 40–60 years remained moderate and that of individuals aged >60 years were rare. A reduction in inter-model variability was observed. Notably, the anesthesia resident and intensive care fellow roles no longer showed statistically significant differences (*p* = 0.059 and *p* = 0.173, respectively), suggesting partial normalization in age representation across models. Detailed results are presented in Supplementary Material S3.

In the grouped analysis, Caucasian ethnicity remained the most frequently represented group across all professional roles and AI models. Inter-model differences decreased compared with previous findings. Minor increases in non-Caucasian representations were observed, particularly in images generated by Gemini and Leonardo, although Caucasian predominance remained unchanged. All roles still showed statistically significant differences among models (*p* < 0.05), indicating that model-level variability in ethnic representation persisted.

## Discussion

This study systematically evaluated the demographic representation of healthcare professionals in anesthesiology, pain medicine, and intensive care across four widely used text-to-image AI models. The findings revealed consistent patterns of bias in sex, ethnicity, skin tone, and professional hierarchy. Male, light-skinned, and Caucasian depictions predominated across nearly all roles, with the most pronounced homogeneity observed in senior leadership positions. These results align with concerns raised in previous studies, indicating that generative AI models tend to reproduce, and in some cases amplify, existing societal stereotypes.^3,5,6^

AI has rapidly evolved from a conceptual technology to an increasingly important component of modern healthcare.^9^ Its applications have expanded from diagnostic support and clinical decision making to educational visualization and communication.^1,2^ In parallel with this expansion, discussions surrounding bias, fairness, and transparency have become increasingly prominent. As AI systems are adopted in sensitive domains such as medicine, the risk that algorithmic decisions or representations may reproduce social inequities has drawn growing ethical concern.^10–12^ This tension between the promise of innovation and the possibility of unintended bias has transformed AI from a purely technical tool into a subject of ethical and professional debate.

Among the various forms of AI, large language models generate human-like text, whereas text-to-image systems convert linguistic prompts into visual content. Both architectures rely on large datasets of human-generated data, making them inherently susceptible to the biases embedded in those sources. Thus, AI systems may replicate existing patterns of bias present in the training data.^13–16^ Exploring these dynamics is particularly relevant in medicine, where professional authority, trust, and identity are often communicated visually. By examining how text-to-image models depict medical professionals, our study aimed to highlight this intersection between algorithmic output and human bias, offering a framework to better understand and eventually mitigate representational inequality in AI-generated imagery.

The concerns surrounding bias in AI became evident in the visual patterns observed in this study. Although the distribution of representations varied by professional role and AI model, the overall trend was consistent. Males were disproportionately depicted, particularly in senior positions. Individuals in leadership roles such as department heads were almost uniformly depicted as male, reflecting persistent cultural associations between authority and masculinity. Although leadership positions in some healthcare settings remain male-dominated, the nearly uniform male representation observed in our results likely represents an overestimation rather than an accurate reflection of the evolving healthcare workforce. Conversely, technical or assistant roles displayed inconsistent sex patterns across models, suggesting that some algorithms encode conflicting assumptions rather than achieving neutrality. This observation may also reflect broader societal patterns in which female representation has traditionally been higher in caregiving and support-oriented professions. These stereotypes may influence how AI systems depict different professional roles.

These findings illustrate how generative AI systems, despite their perceived objectivity, replicate the sex hierarchies embedded in the data from which they learn. Similar patterns have been reported in previous studies, wherein AI-generated imagery in anesthesiology, pharmacy, and paramedicine have overrepresented males and underrepresented females.^5,6,13,17^ Collectively, these results indicate that visual bias in AI is not confined to one field or model but reflects a broader structural tendency to reproduce entrenched social stereotypes. Similar demographic patterns have been reported in previous text-to-image studies, suggesting consistency across different models.^5,6,13,17^

Few previous studies have examined age bias in AI-generated healthcare imagery.^5,6,17^ Our results indicate that younger age groups (<40 years) were most frequently represented, whereas individuals aged >60 years were rarely depicted except in certain leadership roles. This may reflect the intersection of societal perceptions of authority with stereotypical expectations of youth in active clinical roles. Given the lack of comparable age-specific data in previous literature, our study expands the understanding of bias in this dimension. Our analysis revealed a predominance of light skin tones and Caucasian ethnicity across all roles and models, with minimal representation of darker skin tones and non-Caucasian ethnic groups. Comparable studies have also reported similar trends, with DALL·E 3 depicting pharmacists predominantly as White and light-skinned and first responders represented in over 90% of cases as Caucasian according to the terminology used in the respective studies.^5,17,18^ These consistent findings indicate that generative AI systematically underrepresents ethnic diversity, reinforcing narrow professional stereotypes. Similar trends have also been reported across different geographic settings.^6,9^

When images were generated in groups rather than individually, a general attenuation of representational bias was observed across all evaluated dimensions, including sex, age, ethnicity, and skin tone. The grouped generations demonstrated lower inter-model variability, and several previously significant differences became non-significant, particularly in the sex and age categories. However, the overall direction of bias remained unchanged; light-skinned, young, and predominantly Caucasian male representations continued to dominate across approximately all models and professional roles. Although Leonardo and Gemini produced slightly more balanced outputs in terms of sex and ethnicity, DALL·E and Midjourney maintained strong homogeneity toward lighter, male-dominated profiles. Despite the expectation that group-image generation might enhance representational diversity by reducing random bias, this approach did not yield substantially improved performance. Thus, although grouped prompting modestly reduced statistical variability, it failed to overcome the consistent demographic imbalance that characterized the single-image outputs. The use of group-based prompts may have encouraged more generalized or template-like outputs across models, which may have contributed to the observed reduction in representational variability.

A key contribution of this work lies in examining how AI models depict different hierarchical roles within the same specialty. Previous literature has typically focused on a single role or specialty, whereas our analysis spanned 10 distinct positions from trainees to department heads. We observed that biases in sex, ethnicity, and skin tone intensified with increasing professional rank. This gradient suggests that AI models are not only replicating demographic stereotypes but are also embedding hierarchical biases—an aspect largely absent from previous investigations. Although all four AI models demonstrated similar bias patterns, subtle differences emerged. Some models, for example, produced slightly more balanced sex ratios in junior roles or depicted a broader range of skin tones in certain contexts.

Nevertheless, no model consistently outperformed the others in reducing bias across all demographic categories. This reinforces findings from other comparative analyses, demonstrating that bias mitigation remains inconsistent across generative platforms. Recent studies, including that by Gisselbaek et al., have suggested that bias patterns vary across model versions, indicating that ongoing model development influences demographic representation over time.^19^

This study has certain limitations that should be considered when interpreting the findings. The prompts were standardized and prepared in English, which may not have fully captured cultural or linguistic variations in AI-generated outputs. In addition, the study focused on three closely related specialties, which limits the generalizability of the findings to other fields of healthcare. Professions traditionally associated with female representation, such as nursing, were not included in the present analysis; thus, their inclusion in future studies may provide additional insight into sex-associated representation patterns in AI-generated imagery. Our analyses were compared against patterns reported in the literature rather than actual healthcare workforce datasets, representing another important limitation. Furthermore, reverse image search was not performed to verify the originality of AI-generated images, and since some text-to-image generators may produce identical or near-identical outputs when similar prompts are used, this may have influenced image diversity and representation patterns, as noted in previous studies evaluating text-to-image generators for medical illustration.^20^ Although the browser session was refreshed before each generation attempt, the use of the same user account and identical prompts throughout the study may still have contributed to similarities between generated outputs because of the inherent characteristics of generative AI systems. Ethnicity classifications were based on visually defined categories, including the term “Caucasian,” which may not fully reflect the complexity of ethnic diversity and should therefore be interpreted cautiously. In addition, although overall agreement between reviewers was high, category-specific inter-rater agreement values were not calculated separately, and some variability may have occurred in visually subjective categories such as skin tone and age group. Future studies may benefit from incorporating structured evaluation of these features and reporting detailed inter-rater reliability metrics for each variable.

Nevertheless, the potential benefits of AI-generated imagery should not be overlooked. Fan et al. have reported that AI-generated images may help augment rare datasets and support the development of more inclusive visual materials.^21^ Notably, although biases in text-to-image systems may be reduced using more diverse training data and transparent model development, these systems are not inherently resistant to bias.

## Conclusions

This study found that leading text-to-image AI models consistently reproduce images with demographic and hierarchical biases in depictions of anesthesia, pain medicine, and intensive care professionals, with males, light-skinned individuals, and individuals perceived as Caucasian disproportionately represented, especially in leadership roles. By demonstrating that such biases extend beyond demographics to professional rank, our findings highlight the urgent need for more diverse training datasets, transparent model development, and integrated bias mitigation strategies to ensure that AI-generated visuals promote rather than undermine diversity and inclusion in healthcare.

## Supplemental Material

sj-docx-1-imr-10.1177_03000605261470625 - Supplemental material for Visual representation bias in artificial intelligence–generated depictions of anesthesia, pain, and intensive care professionals Supplemental material, sj-docx-1-imr-10.1177_03000605261470625 for Visual representation bias in artificial intelligence–generated depictions of anesthesia, pain, and intensive care professionals by Muzeyyen Beldagli, Ahmet Ozan Aydin, Mehmet Alperen Avci, Selma Kahyaoglu, Taner Tunc, Yavuz Yigit and Serkan Tulgar in Journal of International Medical Research

sj-docx-2-imr-10.1177_03000605261470625 - Supplemental material for Visual representation bias in artificial intelligence–generated depictions of anesthesia, pain, and intensive care professionals Supplemental material, sj-docx-2-imr-10.1177_03000605261470625 for Visual representation bias in artificial intelligence–generated depictions of anesthesia, pain, and intensive care professionals by Muzeyyen Beldagli, Ahmet Ozan Aydin, Mehmet Alperen Avci, Selma Kahyaoglu, Taner Tunc, Yavuz Yigit and Serkan Tulgar in Journal of International Medical Research

sj-docx-3-imr-10.1177_03000605261470625 - Supplemental material for Visual representation bias in artificial intelligence–generated depictions of anesthesia, pain, and intensive care professionals Supplemental material, sj-docx-3-imr-10.1177_03000605261470625 for Visual representation bias in artificial intelligence–generated depictions of anesthesia, pain, and intensive care professionals by Muzeyyen Beldagli, Ahmet Ozan Aydin, Mehmet Alperen Avci, Selma Kahyaoglu, Taner Tunc, Yavuz Yigit and Serkan Tulgar in Journal of International Medical Research
