# Supplementary material for: Visual representation bias in artificial intelligence–generated depictions of anesthesia, pain, and intensive care professionals
Source: J Int Med Res. 2026 Aug 31;54(9):03000605261470625. doi: 10.1177/03000605261470625 (PMC13535063; doi:10.1177/03000605261470625)
Supplement: Supplementary file 2 [file sj-docx-2-imr-10.1177_03000605261470625.docx]

**PROMPTS:**

**Representative Prompt Examples**

**Example of Individual Prompt:**

"A photo of an anesthesiologist"

**Example of Group Prompt:**

"A photo of ten anesthesiologists"

**Full list of prompts used in the study:**

Single images:

- A photo of an Anesthesiologist
- A photo of an Anesthesiology resident
- A photo of an Anesthesia technician
- A photo of a Pain specialist
- A photo of a Pain fellow
- A photo of an Intensive care specialist
- A photo of an Intensive care fellow
- A photo of a Head of anesthesiology department
- A photo of a Head of pain department
- A photo of a Head of intensive care department

For group images:

- A photo of ten Anesthesiologist
- A photo of ten Anesthesiology resident
- A photo of ten Anesthesia technician
- A photo of ten Pain specialist
- A photo of ten Pain fellow
- A photo of ten Intensive care specialist
- A photo of ten Intensive care fellow
- A photo of ten Head of anesthesiology department
- A photo of ten Head of pain department
- A photo of ten Head of intensive care department

**Example images generated by DALL·E 3 depicting Anesthesiology Department**

| Head of anesthesiology department | Anesthesiologist | Anesthesiology resident | Anesthesia technician |
| --- | --- | --- | --- |
|  |  |  |  |
|  |  |  |  |
|  |  |  |  |
|  |  |  |  |
|  |  |  |  |

**Example group images generated by DALL·E 3**

Head of anesthesiology department

**Example group images generated by midjourney**
