# Supplementary material for: Visual representation bias in artificial intelligence–generated depictions of anesthesia, pain, and intensive care professionals
Source: J Int Med Res. 2026 Aug 31;54(9):03000605261470625. doi: 10.1177/03000605261470625 (PMC13535063; doi:10.1177/03000605261470625)
Supplement: Supplementary file 3 [file sj-docx-3-imr-10.1177_03000605261470625.docx]

| Staff | Gender (n) | | | | | | | | χ^2^ | p |
| --- | --- | --- | --- | --- | --- | --- | --- | --- | --- | --- |
|  | Female | | | | Male | | | |  |  |
|  | Dalle | Gemini | Leonardo | Midjourney | Dalle | Gemini | Leonardo | Midjourney |  |  |
| Anesthesia resident | 41 | 43 | 58 | 51 | 61 | 75 | 43 | 42 | 13.868 | 0.003 |
| Anesthesia specialist | 43 | 36 | 50 | 43 | 58 | 76 | 60 | 51 | 5.492 | 0.139 |
| Anesthesia technician | 50 | 50 | 73 | 63 | 53 | 63 | 5 | 21 | 62.345 | <0.001 |
| Head of anesthesia department | 31 | 36 | 34 | 36 | 70 | 78 | 37 | 47 | 8.203 | 0.042 |
| Head of intensive care department | 41 | 29 | 81 | 28 | 59 | 81 | 20 | 52 | 69.574 | <0.001 |
| Head of pain department | 35 | 36 | 66 | 37 | 65 | 81 | 56 | 41 | 16.363 | 0.001 |
| İntensive care fellow | 50 | 42 | 90 | 44 | 56 | 68 | 9 | 41 | 67.240 | <0.001 |
| İntensive care specialist | 45 | 40 | 69 | 51 | 55 | 76 | 29 | 29 | 33.955 | <0.001 |
| Pain fellow | 47 | 49 | 63 | 44 | 57 | 65 | 55 | 47 | 2.822 | 0.420 |
| Pain specialist | 44 | 44 | 53 | 35 | 55 | 69 | 60 | 56 | 2.240 | 0.524 |

**Supplementary Table 1.** Distribution of gender across AI models and professional roles in grouped image analysis. Values represent the number of individuals identified in the generated images. Because AI models did not consistently generate the requested number of individuals per image, totals vary across models and professional roles. Overall comparisons were performed using chi-square tests. **Post hoc pairwise comparisons were conducted using Bonferroni-adjusted chi-square tests.**

| Staff | Skin Tone | | | | | | | | | | | | χ^2^ | p |
| --- | --- | --- | --- | --- | --- | --- | --- | --- | --- | --- | --- | --- | --- | --- |
|  | Light | | | | Medium | | | | Dark | | | |  |  |
|  | Dalle | Gemini | Leonardo | Midjourney | Dalle | Gemini | Leonardo | Midjourney | Dalle | Gemini | Leonardo | Midjourney |  |  |
| Anesthesia resident | 67 | 23 | 40 | 59 | 20 | 92 | 61 | 33 | 15 | 3 | 3 | 1 | 100.888 | <0.001 |
| Anesthesia specialist | 83 | 43 | 103 | 80 | 9 | 69 | 5 | 14 | 11 | 0 | 5 | 0 | 148.301 | <0.001 |
| Anesthesia technician | 72 | 45 | 64 | 69 | 12 | 68 | 7 | 14 | 20 | 0 | 7 | 1 | 119.854 | <0.001 |
| Head of anesthesia department | 82 | 55 | 25 | 38 | 8 | 59 | 45 | 45 | 12 | 0 | 3 | 0 | 83.696 | <0.001 |
| Head of intensive care department | 84 | 45 | 65 | 31 | 8 | 62 | 36 | 48 | 8 | 3 | 2 | 2 | 71.685 | <0.001 |
| Head of pain department | 75 | 70 | 60 | 62 | 13 | 46 | 62 | 16 | 12 | 1 | 4 | 0 | 60.111 | <0.001 |
| Intensive care fellow | 57 | 23 | 31 | 60 | 30 | 87 | 49 | 25 | 20 | 0 | 20 | 1 | 106.297 | <0.001 |
| Intensive care specialist | 74 | 28 | 83 | 48 | 9 | 87 | 11 | 16 | 17 | 2 | 9 | 17 | 161.38 | <0.001 |
| Pain fellow | 58 | 60 | 78 | 59 | 29 | 51 | 36 | 32 | 17 | 3 | 5 | 0 | 35.235 | <0.001 |
| Pain specialist | 64 | 44 | 103 | 63 | 22 | 67 | 9 | 24 | 12 | 2 | 1 | 4 | 95.314 | <0.001 |

**Supplementary Table 2.** Distribution of skin tone categories across AI models and professional roles in grouped image analysis. Skin tone categories were defined according to the Massey–Martin Skin Color Scale as light (1–3), medium (4–7), and dark (8–10). Values represent the number of individuals identified in the generated images. Because AI models did not consistently generate the requested number of individuals per image, totals vary across models and professional roles. Overall comparisons were performed using chi-square tests, followed by Bonferroni-adjusted post hoc pairwise comparisons.

| Staff | Ethnicity | | | | | | | | χ^2^ | p |
| --- | --- | --- | --- | --- | --- | --- | --- | --- | --- | --- |
|  | Caucasian | | | | Non-Caucasian | | | |  |  |
|  | Dalle | Gemini | Leonardo | Midjourney | Dalle | Gemini | Leonardo | Midjourney |  |  |
| Anesthesia resident | 61 | 17 | 40 | 51 | 41 | 101 | 64 | 42 | 57.215 | <0.001 |
| Anesthesia specialist | 79 | 19 | 103 | 78 | 24 | 93 | 10 | 16 | 169.467 | <0.001 |
| Anesthesia technician | 69 | 39 | 64 | 68 | 35 | 74 | 14 | 16 | 63.814 | <0.001 |
| Head of anesthesia department | 82 | 52 | 25 | 27 | 20 | 62 | 48 | 56 | 55.943 | <0.001 |
| Head of intensive care department | 82 | 33 | 65 | 29 | 18 | 77 | 38 | 52 | 70.970 | <0.001 |
| Head of pain department | 72 | 69 | 60 | 39 | 28 | 48 | 66 | 39 | 15.450 | 0.0015 |
| İntensive care fellow | 54 | 19 | 31 | 60 | 53 | 91 | 69 | 26 | 63.251 | <0.001 |
| İntensive care specialist | 74 | 25 | 83 | 43 | 26 | 92 | 20 | 38 | 95.691 | <0.001 |
| Pain fellow | 54 | 45 | 78 | 55 | 50 | 69 | 41 | 36 | 17.775 | <0.001 |
| Pain specialist | 63 | 27 | 103 | 59 | 36 | 86 | 10 | 32 | 108.981 | <0.001 |

**Supplementary Table 3.** Distribution of ethnicity categories across AI models and professional roles in grouped image analysis. Values represent the number of individuals identified in the generated images. Because AI models did not consistently generate the requested number of individuals per image, totals vary across models and professional roles. Overall comparisons were performed using chi-square tests, followed by Bonferroni-adjusted post hoc pairwise comparisons.

| Staff | Age (years) | | | | | | | | | | | | χ^2^ | p |
| --- | --- | --- | --- | --- | --- | --- | --- | --- | --- | --- | --- | --- | --- | --- |
|  | <40 | | | | 40 - 60 | | | | >60 | | | |  |  |
|  | Dalle | Gemini | Leonardo | Midjourney | Dalle | Gemini | Leonardo | Midjourney | Dalle | Gemini | Leonardo | Midjourney |  |  |
| Anesthesia resident | 102 | 118 | 100 | 93 | 0 | 0 | 4 | 0 | 0 | 0 | 0 | 0 | 12.140 | 0.059 |
| Anesthesia specialist | 39 | 86 | 15 | 39 | 57 | 26 | 87 | 52 | 7 | 0 | 11 | 3 | 97.779 | <0.001 |
| Anesthesia technician | 78 | 109 | 61 | 56 | 26 | 4 | 17 | 28 | 0 | 0 | 0 | 0 | 30.482 | <0.001 |
| Head of anesthesia department | 5 | 32 | 20 | 24 | 82 | 74 | 51 | 59 | 15 | 8 | 2 | 0 | 37.254 | <0.001 |
| Head of intensive care department | 11 | 32 | 6 | 10 | 73 | 73 | 90 | 70 | 16 | 5 | 7 | 1 | 41.464 | <0.001 |
| Head of pain department | 16 | 7 | 17 | 31 | 73 | 104 | 101 | 44 | 11 | 6 | 8 | 3 | 44.355 | <0.001 |
| İntensive care fellow | 104 | 110 | 95 | 86 | 3 | 0 | 5 | 0 | 0 | 0 | 0 | 0 | 9.004 | 0.173 |
| İntensive care specialist | 69 | 92 | 22 | 60 | 21 | 25 | 77 | 20 | 10 | 0 | 4 | 1 | 111.868 | <0.001 |
| Pain fellow | 99 | 108 | 74 | 91 | 5 | 6 | 40 | 0 | 0 | 0 | 5 | 0 | 91.258 | <0.001 |
| Pain specialist | 53 | 85 | 4 | 59 | 43 | 28 | 95 | 32 | 3 | 0 | 14 | 0 | 144.439 | <0.001 |

**Supplementary Table 4.** Distribution of age groups across AI models and professional roles in grouped image analysis. Age categories were defined as <40 years, 40–60 years, and >60 years. Values represent the number of individuals identified in the generated images. Because AI models did not consistently generate the requested number of individuals per image, totals vary across models and professional roles. Overall comparisons were performed using chi-square tests, followed by Bonferroni-adjusted post hoc pairwise comparisons.
